# Human capabilities of South African parents who have children with developmental disabilities

**DOI:** 10.4102/ajod.v12i0.1155

**Published:** 2023-06-19

**Authors:** Lumka Magidigidi, Nicolette V. Roman, Inge K. Sonn

**Affiliations:** 1 Centre for Interdisciplinary Studies of Children, Families and Society, Faculty of Community and Health Sciences, University of the Western Cape, Cape Town, South Africa

**Keywords:** human capabilities, freedoms, opportunities, developmental disability, parents, children with disabilities, family, capabilities approach, South Africa

## Abstract

**Background:**

Parenting a child with a developmental disability (DD) has a substantial influence on the lives of the parents or caregivers, as well as on how the family operates. This is frequently because of the adjustments in some daily practices that are crucial for parents’ or caregivers’ human capabilities to provide for childcare. There is not enough research done on human capabilities of parents or children with DD in South Africa.

**Objectives:**

This study investigated the available support in improving the human capabilities of parents or caregivers with children with DD and the bodily health and bodily integrity human capabilities of parents or caregivers with children with DD.

**Method:**

Qualitative interviews were conducted with 11 parents or caregivers of children aged between 1 and 8 years old with DD. This study used snowball sampling. Thematic data analysis was chosen to analyse the data collected.

**Results:**

The results of the study indicate that participants have difficulties bringing up their children because of the emotional strain that goes along with parenting a child with DD. In addition, participants were not able to afford decent and satisfactory shelter and had limited access to good quality food because they could not afford it.

**Conclusion:**

A lack of social support and care burden influences parents’ or caregivers’ ability to raise their child with developmental disability.

**Contribution:**

The study contains helpful information about families of children with DD in under-resourced locations. The information may be of significance to policymakers who are accountable for designing and executing policies that are targeted at assisting parents or caregivers of children with DD.

## Introduction

According to the World Health Organization (WHO) (2018), globally, around 1 billion people live with a disability and about 200 million children are likely to be living with some type of disability. Under the WHO (2018), high-income countries have a child disability rate of 2.8%, which is higher than the global rate of 5.0%. As defined by Olusanya et al. (2018), developmental disabilities are a group of conditions stemming from impairments that affect a child’s physical, learning, or developmental performance. Affected children generally have sensory impairments such as epilepsy or seizures, intellectual disability, cerebral palsy, attention deficit hyperactivity disorder (ADHD), autism spectrum disorder (ASD), hearing and vision loss, or more learning disorders. Findings from Zablotsky et al. (2019) indicated that the prevalence of developmental disability (DD) among U.S. children aged 3–17 years increased between 2009 and 2017. The National Health Interview Survey (NHIS) indicated that from 2009 to 2017, there was a 9.5% increase in the prevalence of developmental disabilities among children aged 3–17. The prevalence of any DD increased significantly by 16.22% to 17.76%, which indicated a rise of 9.5% when comparing the years from 2009–2011 to 2015–2017. In this period, noteworthy increases were also observed for ADHD (8.47% to 9.54%, with an increase of 12.6%), ASD (1.12% to 2.49%, with an increase of 122.3%), and intellectual disability (ID) (0.93% to 1.17%; with an increase of 25.8%), nevertheless, a considerable decrease was seen for the category of ‘other developmental delay’ (4.65% to 4.06%; a decrease of 12.7%).

In addition, the International Classification of Functioning, Disability, and Health (ICF) defines disability as impairments that limit the movement, activity, participation and engagement that result from the interaction between the environment of the person impacted by the health condition (Schiariti, Mahdi & Bölte 2018). As a result of its ICF, the WHO acknowledges that disability is not limited to a deviation from a basic standard; therefore, it is not an individual characteristic, but rather multilayered interaction among individuals with impairments and contextual circumstances such as poverty (Moreno, Bennett & Ferrite 2022). For this article, poverty is defined as a condition characterized by severe scarcity of basic human needs, considering food, safe drinking water, sanitation facilities, health, housing, education and information. It depends not only on income but also on gaining access to services (United Nations 1995). These aspects may have an impact on the bodily and emotional health of parents or caregivers and their capability to satisfy their child’s developmental requirements (Benn et al. 2012). Parents or caregivers of DD children may also suffer from poor health as a result of poverty and access to or unavailability of healthcare services and assistive devices (Geere et al. 2013). It is also common for parents or caregivers with children with developmental disabilities to experience poverty and scarcity or lack of economic assistance, to lack reasonable access to the necessary information to provide appropriate maintenance to their children, and to have insufficient social support (DSD, DWCPD & UNICEF 2012). Deprivation, such as poverty, overcrowded housing and unemployment, also affects parents’ ability to care for their children adequately (Ward, Brown & Hyde-Dryden 2014). Moreover, Gupta, Featherstone and White (2016) added that children’s well-being can be promoted by government assistance to their parents or caregivers, and by improving the living standards of poor families, including improving public housing, schools and other services.

In the light of this, the South African government has acknowledged that poverty has a significant effect on children with severe disabilities (DSD et al. 2012). Assistive devices or physiotherapy may be needed for children with disabilities. They may also require special attention, medication and ongoing treatment. Families already struggling to make ends meet could be further burdened by these additional costs (ACPF 2011). To support the country’s overall growth, the government developed policies promoting political, social and economic transformation (ACPF 2011). One of these policies was mentioned by Tigere and Makhubele (2019), who pointed out that to alleviate poverty, South Africa heavily invests in policies that ensure that all parts of the population have access to basic care. In addition, they have access to appropriate facilities such as housing, sanitation and energy sources (Richardson 2018).

Caregiving a child with a DD is therefore difficult because of the child’s impairments and comorbidities, social contexts, the child’s extensive presentation of disability, and the absence of support systems (Zhao & Fu 2022). In addition, children with impairments are particularly affected negatively by poor living conditions (McKinney et al. 2021). According to Statistics South Africa (STATSSA) Community Survey 2007, compared with their counterparts without disabilities, children with disabilities are less likely to have access to proper housing, water and sanitation (Visser et al. 2016). Contrary to those who live in traditional homes and informal settlements, children with disabilities are likely to have more opportunities and access to support in higher socioeconomic circumstances (McKinney et al. 2021). Children with disabilities and their families are put under a great deal of stress by overcrowded living situations and outside public toilets (DSD & UNICEF 2012). This, however, has been identified by Mörelius and Hemmingsson (2014) to affect the parents or caregivers taking care of the child.

Therefore, this study used the Human Capabilities Approach to explore the human capabilities of South African parents or caregivers of children with DD. Nussbaum (2011) developed the notion of human capabilities to think about social justice and the fundamental duties of a state to its citizens. Furthermore, the various circumstances that surround people’s lives have an impact on their ability to perform (Nussbaum 2011). In this case, competent prospective parents who have completed their secondary or tertiary education may be more equipped to assist their children’s education at home and foster an environment that is conducive to their academic achievement (Hartas 2014).

### Disability and Nusbaum’s capability approach

Disability, according to the WHO (2018), is a general term for impairments, movement restrictions and participation restrictions. The term is used to describe a person’s performance as well as bodily, intellectual, physical and mental health, as well as various forms of lifelong disabilities (WHO 2018). Fredman (2017) defines disability as an impairment that affects the body (functioning and structure); activities and participation; and contextual factors (social factors that may affect performance). However, Mitra (2017) defines a disability as a deprivation related to functioning and/or capability among persons who require good health. Rather than taking into account merely the right or freedom of individuals to pursue their well-being, Nussbaum (2011) defines a capability approach to human welfare as focusing on the actual capacity of individuals to achieve their well-being. As Nussbaum explains, ‘capabilities’ are the conditions or states of accomplishment that enable people to accomplish things such as moving freely between places. It is also very important that people have the freedom to live the kind of lives they desire, to do what they want, and to be the individuals they desire because that is what makes them great. Based on Nussbaum’s (2000) capability approach, parents and caregivers with children with DD experience differing levels of functioning and interactions based on their living conditions and their ability to meet the needs of their children.

According to the WHO (2012), children can become more serious and have lifelong effects, increase deprivation, and be more excluded from society if they are not offered early intervention, assistance and security, on time. A paucity of literature exists on parenting or caring for a child with DD based on human capabilities. As a result, a global research study by the United Nations International Children’s Emergency Fund (UNICEF 2011) suggested that states should offer assistance, support and services to parents of children with DD so they can care for and raise their children.

Similarly, UNICEF (2011) stated that developing nations have a responsibility to provide parents and carers of children with disabilities with the necessary support so that they can care for their children. UNICEF (2011) adds that nations should provide early knowledge, provisions and assistance to children with disabilities and their families to prevent repression, rejection, mistreatment and exclusion. According to UNICEF (2011), children with impairments continue to have equal rights in the home. Because of this, people with disabilities frequently have more healthcare demands than others, such as those related to basic health disorders and impairments, disability screening and the treatment of infections (Beurkens et al. 2013). As a result, the United Nations (2018) said that people with disabilities incur costs for things such as healthcare, transportation, specialized aids or gadgets, and house modifications to accommodate the child’s condition, among other things. It has been established that having access to financial services is crucial for helping people escape poverty (McKinney et al. 2021). As a result of poverty or unemployment, parents may find it difficult to upgrade their skills, purchase a home or pay for their children’s education (United Nations 2018).

### South African policies for children with disabilities

As a co-signatory to both the United Nations Convention on the Rights of the Child (UNCRC) in 1995 and the United Nations Convention on the Rights of Persons with Disabilities (UNCRPD) in 2007, South Africa has some of the best policies for children with disabilities (Tigere & Makhubele 2019). However, statistics from STATSSA (2014) showed that children with disabilities continue to be less likely than their without disability counterparts to have access to decent housing, clean water, and sanitary conditions. Furthermore, research shows that children with disabilities are more likely than their without disability peers to live in outmoded homes in unconstitutional neighbourhoods. Families and people with disabilities are under a lot of stress because of overcrowded housing conditions and outdoor restrooms (DSD et al. 2012). As a result, it is crucial to take into account the parent’s ability to raise these children because they are so fundamental to the development of these kids.

### Parenting children with a disability within capability’s approach

As stated by Gupta et al. (2016), functioning’s are identified as objects or activities that people are interested in as well as estimated states of being, such as prosperity and well-being of individuals. Opportunities to carry out such functions are known as capabilities (Mitra 2006). The person’s capabilities depend on factors that are under their control, such as their physical qualities, financial situation and degree of education, as well as their socio-political background, which can either increase or decrease their capabilities. In response to the capability approach, parents’ functioning that is what they accomplish in their interactions with children is constrained by their living circumstances and their capacity to turn opportunities into functioning (i.e. their access to and ability to take advantage of real opportunities) (Hartas 2014).

Therefore, the study aimed to explore the human capabilities of parents or caregivers with children with DD.

The two main objectives of the study are: (1) to explore the bodily health and bodily integrity capabilities of parents or caregivers of children with DD and (2) to explore how the government assists parents or caregivers of children with DD in enhancing the human capabilities of parents.

## Research methods and design

### Study design

A qualitative research approach was employed to explore the human capabilities of parents or caregivers of children with developmental disabilities, between the ages of 1 and 8 years old. This method allowed the researcher to create a greater understanding of human capabilities, including parents or caregivers freedoms and their bodily health and bodily integrity (Safe, Joosten & Molineux 2012). According to this study, parents or caregivers’ physical and mental well-being are related to their bodily health. Contrarily, bodily integrity refers to parents or caregivers freedom to move around and the right to autonomy as well as the absence of outside prejudice. Qualitative research was appropriate for this study because, as Clarke and Braun (2013) suggested, it illustrates the difficulty, confusion and contradiction that characterize reality while still enabling us to make sense of many types of meaning. An exploratory-descriptive research design was employed to explore new points of view or ideas, about the human capabilities of parents or caregivers with children with developmental disabilities (Ali et al. 2017). The researcher was able to build interpretations from the perspectives of participants using this form of study design without having any preconceived notions and to gain a personal grasp of the research problem (Krysik & Finn 2010:309). As mentioned by Ali et al. (2017), a descriptive research design is a technique that, after data have been gathered from a specific sample, provides details of the characteristics of the study population. An exploratory research approach reveals a topic’s key characteristics and its relevance to the study. Therefore, in this study, the focus was on the human capabilities of parents or caregivers with children with developmental disabilities and explored the bodily health and bodily integrity capability of parents or caregivers of children with developmental disabilities.

### Research setting

The study was carried out in two distinct townships in the Western Province of Cape Town, namely the Kraaifontein and Fisantekraal locations.

Kraaifontein is a township of 154 615 citizens and just 5% of these people have a college degree. Kraaifontein contains 40 169 homes, 33.2% of which are headed by women. Kraaifontein consists of 49.8% of men and 50.2% of women make up the population. Most of its residents (43.3%) are black African people. Furthermore, a variety of races can be found in the area, including white people (14.4%), any other races (1.7%), and Indian people and Asian people (0.4%), according to STATSSA (2014).

The second research setting area was Fisantekraal location. Fisantekraal is a township with a total population of 12 369 people, 50% of whom are men and 49% of whom are women. There are 3712 homes in this township and 35% of them are headed by women. A total of 1.1% of the population of Fisantekraal has received higher education. The area consists of 52% of piped water inside a dwelling and 68.5% of the households in the area have electricity. Black people make up 51.5% of the population followed by mixed-race people (46.9%), Asian people and Indian people (0.3%), and other races (0.8%).

### Study population and sampling strategy

The sample was drawn from a population that met the criteria of the study: (1) they are caregivers or parents of children with DD and (2) who reside in Kraaifontein and Fisantekraal locations in Cape Town. A non-probability purposive sampling of 11 parents or caregivers of children with DD who had been chosen to participate in the study was used in the study. Snowball sampling, a kind of purposive sampling also known as chain referral sampling, was used in this investigation. Using this method, the participants or informants who interacted with the researcher used their social connections to connect with more parents or caregivers of children with DD who participated in the study.

Participants in the study sample were more likely to be women than men. The ages of the 11 study participants who took part ranged from 20 to 59. The majority of participants mentioned that they were unemployed at the time of data collection. However, from the 11 participants, there were only two participants who were working or employed at the time of data collection.

### Data collection

Semi-structured in-person interviews with the guidance of an interview guide were used to gather the data. With the participants’ consent, all of the interviews were audio recorded in their selected language. Only one participant preferred an interview in English, while the majority of participants requested that their questions be answered in isiXhosa. The interviews took place in the comfort of the interviewees’ homes and lasted between 30 and 40 min each. The researcher provided a brief overview of the study to all 11 participants while reading and expanding the informed consent form and the information sheet (World Medical Association 2001). Depending on the participant’s preference, either Xhosa or English was used for the reading. Subsequently, the study was further explained, and participants signed a consent form to participate in the study. Furthermore, the researcher notified the participants of their right to voluntarily participate in the study and to discontinue participation at any time. In addition, participants had the chance to ask the researcher any questions they had and the researcher responded. The confidentiality and anonymity of the participants in this study were taken into consideration and it was made extremely obvious to the participants. Ritchie et al. (eds. 2013) put forward that anonymity means the identity of those taking part not being identified in the research group.

### Data analysis

The study used thematic analysis, as highlighted by Nowell et al. (2017) that in qualitative research methods, thematic analysis can be mostly employed through a range of epistemologies and research questions. Thematic data analysis was selected because it allowed the researcher to identify, analyse, categorize, describe and report themes identified in the data set, as alluded to by Braun and Clarke (2006). The study was analysed in six phases of the thematic analytic process: (1) *Familiarising with the data:* Field notes from interviews, participant observations, and a reflective notebook that was kept after each interview were all used as textual data in the study. To assure transparency and gather additional information to provide usable and consistent data, the researcher also applied the idea of triangulation, using a variety of sources of data (Leedy & Ormrod 2014). The study used field notes and interviews as its data collection methods. (2) *Creating themes:* After familiarising themselves with the data, the researchers created themes based on the information gathered. (3) *Generating codes:* The researchers in this case used all of the data that had been gathered, identifying and constructing the analytical building blocks. The approach used in this study was open coding to code sections. Similar codes were joined in this method to create a new code with a wide range of applications. (4) *Reviewing potential themes:* The researchers identified themes from the data that were acquired and ensured that they addressed the main goals and objectives of the investigation. (5) *Defining and naming themes:* According to this perspective, the researcher created an overall narrative of all the data, making sure that each theme was consistent with the thesis’s main plot. (6) *Producing a report:* Each theme has been well supported by the researcher, who used specific examples from the used data when appropriate.

### Ethical considerations

Permission to conduct the research was attained from the Humanities and Social Science Research Ethics Committee (HSSREC) at the University of the Western Cape. An evaluation of ethical standards was performed to safeguard professional practice in the study. This means that the permission permitted the study to adhere to ethical principles designed to determine whether specified actions or developments are right or wrong and giving rules to professionals to avoid professional misconduct was considered (Sarantakos 2012).

## Findings and discussion

The themes chosen for the study were: *body health* (physical, mental, and social health), *bodily integrity* (freedom of movement, freedom from acts of violence), and unrestricted access to healthcare and *governmental assistance*. The themes came about to better understand and explore the human capabilities and sub-themes across two different capabilities. Each capability’s necessary information was divided into distinct sub-themes under each topic (see Table 1). The three themes include: (1) the bodily health of parents or caregivers of children with DD, (2) the bodily integrity of parents or caregivers of children with DD, and lastly (3) the governmental assistance to parents and caregivers of children with developmental disabilities in enhancing the human capabilities of parents were presented as findings for the study.

**TABLE 1:** Themes and sub-themes.

Themes	Sub-themes
Bodily health of parents or caregivers of children with DD	Good health
	Access to good food
	Adequate shelter
Bodily integrity of parents or caregivers of children with DD	Freedom of movement
	Security against violence and discrimination
Governmental assistance to parents or caregivers of children with DD in enhancing the human capabilities of parents or caregivers	Financial assistance
	Counselling

DD, developmental disability.

### Theme 1: Bodily health of parents or caregivers of children with developmental disability

#### Good health

The study’s findings showed that the majority of parents or caregivers understood the value of physical health and had various strategies for ensuring their children’s physical well-being. The term ‘bodily health’ refers to a person’s whole well-being, taking into account all of their physical, mental, social, emotional and spiritual needs for them to thrive rather than just get by Abma et al. (2019). Nussbaum (2000) argues that maintaining physical health involves being able to live a healthy life, having access to medical care when it’s needed, eating a healthy diet, and being able to exercise.

#### Access to good food

In a nutshell, Nussbaum’s second capability is to be in good physical health and this capability advised sustaining health (Magidigidi 2021). One of the participants thought that being healthy was:

‘… eat healthier food which includes fruits and vegetables and some of them have higher protein which will make us live a healthier life.’ (Participant 5, 20-year-old, male)

To sustain good health, participants stated that they:

‘… eat good food not food with too many fats … eating things that will be beneficial for my health.’ (Participant 1, 38-year-old, female)

‘I walk as part of my exercises; walking is a good exercise.’ (Participant 10, 51-year-old, female)

‘For mental health, I usually talk with my neighbor, we go to the same church.’ (Participant 2, 56-year-old, female)

The participants’ response shows that they are aware of what physical health entails. The responses reflect Nussbaum’s (2000) discussion of the importance of eating well, being able to exercise and maintaining health. Based on Nussbaum’s (2011) capabilities approach, this indicates that by emphasising the benefits of being capable of performing healthy acts, the capacity approach may assist in bringing about this change towards healthy eating or active living.

This indicates that parents are equipped with the knowledge they need to live healthy lives and utilize the healthy options at their disposal. This shows that parents or caregivers have the capability for physical health so that they can work to maintain the health of their bodies. Participants in the study have acknowledged the importance of exercising this capability. Access to healthy food, according to Nussbaum (2000), is essential. Participants demonstrated their awareness of healthy eating; however, many claimed that they are undernourished because good food is expensive. This study explored the bodily health capabilities of parents or caregivers of children with DD. Furthermore, as stated by Mörelius and Hemmingsson (2014), parents of children with DD run the risk of having a lower quality of life in terms of their health, particularly regarding daily tasks, sleep, energy, stressful emotions and social interactions.

Emotional difficulties such as depression may have an impact on parents who are overseeing and managing the medical condition of their child. The incapability of the family to deal with the child’s impairment may have a greater impact on the parent’s health because emotional stress in the parent may result in emotional and psychiatric stress in the child (Hung et al. 2010). The results are in line with those of a study by Mörelius and Hemmingsson (2014), which showed that when a child had sleeping issues, both parents or caregivers of a child with a physical disability reported decreased health, emotional exhaustion, staying up later and having interrupted sleep. For example, Participant 2, indicated emotional and physical tiredness, she mentioned that she was:

‘[*U*]sually me alone who was taking care of her, so at times I needed help, even though it is scarce but I do need help like to sometimes help me lift him, you see now as he is laying there, I can’t even though I say I can feed him but I can’t lift him by myself. We end up falling together.’ (Participant 2, 56-year-old, female)

Furthermore, when the child has sleep issues, the parents or caregivers reported more headaches. These incidents demonstrate how difficult it is for parents or caregivers of children with DD to raise their children because of the emotional strain that comes with raising a child with DD (Mörelius & Hemmingsson 2014).

This means that participants are therefore subjected to stress and anxiety as some of them are not even fully knowledgeable regarding the disabilities their children have. Therefore, it is crucial that the government and non-governmental organisations (NGOs) create and strengthen initiatives to assist parents or other caregivers in the Kraaifontein and Fisantekraal locations in the Western Cape province. This will enable the caregivers to cope with their situations and consequently increase their capabilities. Most of the participants stated that they are unemployed (see Table 2). Therefore, there is a need for sustainable livelihood projects in the area to give the participants a way to make money to support themselves and the children under them so they can afford to eat well, stay healthy, and have a place to live.

**TABLE 2:** Demographics of parents or caregivers.

Name	Age	Sex	Employment status	Age of child	Financial support	Type of disability
Participant 1	54	Female	Unemployed	5	Child disability grant	Autistic
Participant 2	56	Female	Unemployed	24	Foster care grant	Cerebral parsley
Participant 3	32	Female	Unemployed	7	Child disability grant	Down syndrome
Participant 4	21	Female	Unemployed	3	Child disability grant	Foetal alcohol syndrome (FAS)
Participant 5	20	Male	Employed	1	Monthly salary	Intellectual disability (fragile x)
Participant 6	30	Female	Unemployed	8	Child disability grant	Spastic cerebral parsley
Participant 7	40	Female	Unemployed	3	Child grant	Autistic
Participant 8	47	Female	Unemployed	5	Child disability grant	Autistic
Participant 9	35	Female	Unemployed	4	Disability grant	Cerebral parsley
Participant 10	51	Female	Unemployed	8	No financial support	Unclassified DD (child cannot talk)
Participant 11	38	Female	Employed	5	Disability grant	Autistic and congenital myotonic dystrophy

The government as well as NGOs working in the Kraaifontein and Fisantekraal areas need to develop and strengthen support groups for parents or caregivers of children with DD. This will assist the caregivers with emotional support from others with whom they share similar situations. Evidence from a study by Ignjatović (2019) also indicates that when parents or caregivers receive more outside support, they can experience some relief that could add to the advancement of social interactions between parents, as well as to their parenting. The parents or caregivers will learn and share their experiences with others. The support groups’ facilitators can also engage experts who can educate the parents or caregivers on how they can care for their children with a particular disability. As a result, the parent or caregiver will have a better understanding of the child’s disability and how to help their child live a more fulfilling life. Psychologists, social workers and paediatricians can all provide expert assistance.

#### Adequate shelter

The study’s findings further demonstrated that participants are unable to provide themselves and their children with an appropriate and adequate place to live. Participants’ findings suggested that the parents or caregivers and children don’t have access to decent homes. This demonstrates that the living situation the participants rely on is not enough and suitable for them and their children. Some participants share a room in a one-room apartment with the children. As a result, a participant clarified this by mentioning:

‘The condition of this house does not accommodate him because our space is small. Because he is a person walking in a wheelchair [sic], he does not have space, so he stays in one place he roams in one place.’ (Participant 9, 35-year-old, female)

While parents were evaluated on their level of health, the majority of participants demonstrated a lack of capability for health, among them one said:

‘I am not emotionally fine. The life that I am living is very painful because other people who have children who do not have disabilities do not live the kind of life that I am living. I feel like I am not living a proper life because most of the time I do not get enough space to be. At times I do think about working but I can’t because of my son’s life. I must look after him. I also have arthritis and it is difficult for me to take my child to the hospital.’ (Participant 1, 54-year-old, female)

Many study participants said they depend on renting a room for themselves and their kids because they do not own a home. Gupta et al. (2016) state that it is obvious that social and environmental factors from both the past and the present have a significant impact on a person’s skills.

### Theme 2: Bodily integrity of parents or caregivers of children with developmental disability

Many of the participants argued that their children’s disability prevents them from moving freely from one place to another.

#### Freedom of movement

Most of the time because of difficulties with mobility concerns, they are unable to travel with their children. The participants also mentioned that they were typically at home all the time because they needed to check on their children frequently to make sure they were not injuring themselves. One of the participants said she was unable to go shopping with her 5-year-old autistic child because of her child’s behavioural problem. She went on to say:

‘… I use public transport because it’s accessible to me and affordable …, trains are not traveling properly in Fisante, they travel badly and another thing is a taxi is quicker than a train, I am that mother that wherever I go I know I must quickly come back. I don’t have the liberty of staying and shopping around. At least a taxi is quicker, I am not saying it’s the best, it’s what is available to me but it is quicker than any other mode of transport that I can use.’ (Participant 8, 47-year-old, female)

According to Nussbaum’s (2000) capabilities perspective, given that parents or caregivers of children with DD are unable to move freely from one location to another as a result of their children’s circumstances, it might be assumed that they lack bodily integrity.

#### Security against violence and discrimination

As stated by Nussbaum and Sen (1993), maintaining one’s physical integrity involves being protected from assault, such as sexual or domestic violence. Particularly, several of the participants claimed that because of the health of their children, they had never experienced abuse or any type of discrimination. Only a small number of participants, although are protected from violence and discrimination. One participant said the following:

‘… many people always have something to say about my child’s disability, I was very young when I got pregnant with her. At first, I didn’t know that I was pregnant and that’s why I drank so much. People now always judge me because my child has this condition.’ (Participant 4, 21-year-old, female)

Another participant indicated that:

‘When arguing with someone who insulted me about my child’s condition, I was pained because she insulted me about my child.’ (Participant 9, 35-year-old, female)

The majority of the participants have been made fun of and given harsh remarks. As an illustration, participant 1 disclosed that:

‘Yes, I encounter discrimination so much, as a result, we have a case that is still pending in court again. People here in the community refer to him in a very painful manner saying that “that kid who is disabled.”’ (Participant 1, 54-year-old, female)

As a result, this demonstrates the necessity for programmes or awareness campaigns to promote social cohesiveness and lessen any ongoing danger, violence or prejudice (DSD et al. 2012; Alter et al. 2013).

### Theme 3: Governmental assistance to parents or caregivers of children with developmental disability in enhancing the human capabilities of parents or caregivers

Under the capabilities approach, the government has a responsibility to care for its citizens (Nussbaum 2000). When asked what kind of support they get from the government or their community, the participants were mostly able to describe the kind of support they get from their immediate surroundings and how it helps them. The majority of interviewees reported that their child’s disability grant was their only source of income or assistance. Others referenced receiving free counselling and boxes of groceries as additional types of community help. One parent mentioned that she uses online support services in addition to the one-on-one assistance she receives from the community and governmental organisations to deal with the situation. Given that parents’ needs differ, the government must find alternative assistance programmes. As an example, some people prefer financial support over others while others find psychological support to be more beneficial. In the light of this, the government needs a wide range of resources to meet the demands of various parents or caregivers.

#### Financial assistance

As a result of the care obligations associated with caring for a child with disability, parents or caregivers may find it difficult to find employment, which could ultimately put them under further financial stress (Muller-Kluits & Slabbert 2018). As a result, this section of the study shows the research findings about the community, governmental or non-governmental organisation (NGO) interventions that are available and intended to help the parents or caregivers of children with DD in the researched area. Participants in the study reported that they are recipients of a disability allowance from the Department of Social Development. The disability grant is designed to make it easier for parents and other carers of children with Down syndrome to meet their needs. Participant 1 remarked:

‘[*crying*], support? …. [*Tears roll down her face and crying more*]. The only support I get from the Government is his grant. Mine is…. When I’m under a lot of pressure, I take a lot of medication you won’t like it when you see my pills (sobbing, talking with a shaky voice) you will not like it see all my pills. I do go to the doctors and they only give me six (6) months’ grant (voice becomes lower and nose blocked from crying) that period is short, and it ends quickly. I then become worried and hear things in my ears, sometimes it would be like I’m awake but I’m not, maybe it is thinking a lot. So it becomes easier when I get grant support for me, at least I know that money I take as my wages and buy food with it, and then the grant money I can buy all his stuff, clothe him, and feed him and his sister and then I can buy things like electricity so that they can both with hot water. With mine, I take it as my wages, when it is depleted like right now it becomes a problem. There is that month that they skip [*sobbing*], you see I become free when I have my grant but when I do not have it pains me a lot.’ (Participant 3, 32-year-old, female)

Food support is one of the strategies or tactics used to help parents and carers of children with disability. This aid is frequently provided by the government through the Department of Social Development. To make sure that the parents or caregivers can feed their children, nutritional assistance is provided. One more participant remarked:

‘Sometimes they call us into the hall and Social Development will distribute food parcels although the food items are small and few. Sometimes you find it will be only 2 kgs of mealie meal [*South African moderately coarse flour made from maize or mealies*]and 750 g of cooking oil but then it is not the same it closes a gap.’ (Participant 8, 47-year-old, female)

#### Counselling

The participants experience stress and worry because some of them are dissatisfied with the help that was given to them. Results from the study conducted by Ignjatović (2019) support the claim that parents of children with disabilities are not receiving the genuine assistance they require. Therefore, the government has to provide NGOs with funding and support so they can create and improve initiatives to help parents or other caregivers in the Kraaifontein and Fisantekraal locations in the Western Cape province. The parent’s or caregivers’ capability will be enhanced as a result of being able to manage their circumstances. The majority of individuals acknowledged being jobless. According to the WHO (2012), interventions including support groups, group conversations and one-on-one listening can give parents of children with similar disabilities time to share experiences and encourage one another. Counselling is a different programme available to parents and carers of children with DD. According to one of the study participants, she seeks counselling anytime she feels overburdened by her circumstances. Her response, which included the following, clarifies this:

‘I do go for counseling when I have a lot going on in my mind … finding out that I had a lot of diseases and the condition of my child made me fearful … but because of consistent counseling, here I am today. I go to Kraaifontein Day Hospital and Tygerberg Hospital for counseling services when I am troubled emotionally.’ (Participant 1, 54-year-old, female)

To improve the well-being of the entire family, Sammon (2018) contends that counselling is crucial for families with children who have difficulties. Counselling is beneficial for parents or caregivers of children with DD, according to one study.

According to published research (Shandra et al. 2008), raising children with disabilities is stressful. To help the parents of children with developmental disabilities, it is necessary to establish effective support groups in the local communities. The National Academies of Sciences, Engineering, and Medicine (2016) also stressed the significance of support groups or programmes because they provide parents of children with disability a chance to come together to discuss shared struggles and worries. As a result, the parent or caregiver would be able to encourage one another by sharing their experiences with others who have children with the same impairment through these support groups.

### Limitations

A small sample of 11 participants, each of whom was interviewed once, made up this study. This implies that some crucial ideas might have been overlooked or improperly developed. The fact that the phenomenon studied cannot be applied to all groups is another drawback. This is so because just a small portion of people receiving disability care were included in the sample, which was also not chosen at random. Limitations in the study were highlighted by the participants’ demographic data, which showed that none of them were both parents or mother and father figure caregivers. The limitations of this study were as follows:

- There was only one primary parent or caregiver (mother or father) accessible for the study; however, the study intended to interview both the mother and father. There would have been a range of knowledge and understanding regarding the human capabilities of parents or caregivers of children with DD had both parents and caregivers or more male parents or caregivers been present during the study. Despite this, the data provided by the participants allowed for data saturation, which appears to adequately convey the human traits and experiences of these parents or caregivers.
- Even though the study did not specifically aim to explore the experiences regarding social class, the findings have shown that almost all the participants were not formally employed because of the disability of the child. Prior research has shown that disability is related to socioeconomic background (poverty), even though the study did not specifically aim to explore the experiences regarding social class.
- Only parents or caregivers of children with DD were included in the study. This indicates that not all parents or caregivers of children with other forms of disabilities took part in the study.

### Recommendations

The survey found that many of the participants are jobless and unable to hunt for work because they must be constantly present at home to care for their dependent children. This study urges for the development of sustainable livelihood initiatives, such as support groups specifically for parents and caregivers of children with disabilities, which can be supported by local NGOs, so that the parents or caregivers can have a reliable source of income to support themselves and their children. This might significantly reduce issues with insufficient food and ill health. As the parents or caregivers will have a source of money, it might help reduce emotional stress.

## Conclusion

The human capability of parents or caregivers of children with DD was investigated in this study. According to the study’s findings, parents or caregivers of children with DD are less capable than those of children without disabilities because of underlying reasons such as unemployment, the responsibility of providing for their needs, and higher levels of stress. The ability of the parents or caregivers to care for their children with disability is negatively impacted by this. Given that people live independently and there is a great need to strengthen the support networks for parents or caregivers. The capabilities of parents or caregivers of children with DD are likely to be improved if there are strong family and community links. Thus, this study comes to the conclusion that there is a need to move away from remedial or welfare assistance and instead put more emphasis on social or communal development methods.
